# Comparison of the efficacy of one-hole split endoscopy and unilateral biportal endoscopy in the treatment of lumbar disc herniation

**DOI:** 10.3389/fsurg.2026.1765537

**Published:** 2026-07-21

**Authors:** Xiaowei Yang, Shimin Li, Lin Xu, Xiaofeng Yang, Bingchuan Xue, Xiao Ouyang, Songyi Ning

**Affiliations:** 1 Yangzhou University Medical College, Yangzhou, Jiangsu, China; 2 Department of Orthopedic, Xuzhou Cancer Hospital, Xuzhou, Jiangsu, China; 3 Department of Orthopedic, Xuzhou New Health Hospital, Xuzhou, Jiangsu, China; 4 Yangzhou University Affiliated Hospital, Xuzhou, Jiangsu, China

**Keywords:** comparison, efficacy, lumbar disc herniation, one-hole split endoscopy, unilateral biportal endoscopy

## Abstract

**Objective:**

The aim of this study is to compare the efficacy of one-hole split endoscopy (OSE) and unilateral biportal endoscopy (UBE) in the treatment of lumbar disc herniation (LDH).

**Methods:**

Retrospective analysis of 101 LDH patients who underwent UBE or OSE surgery in our hospital from January 2022 to January 2024. Among them, 53 cases received UBE treatment, while the remaining patients received OSE treatment. Evaluate patient outcomes, including changes in Hb and Hct levels, incision length, total blood loss (TBL), surgical duration, hospital stay, complications, visual analog scale (VAS) for back and leg pain, Oswestry Disability Index (ODI), and efficacy evaluated using modified MacNab criteria.

**Results:**

After surgery, Hb and Hct levels decreased in both groups; however, the absolute postoperative Hb and Hct levels were significantly higher in the OSE group than in the UBE group (*P* < 0.05). The incision length and TBL in the OSE group were lower than those in the UBE group, but the surgical time was longer than that in the UBE group (*P* < 0.05). There were no significant difference in the incidence of postoperative complications and the excellent rate after 12 months of follow-up between the two groups (*P* > 0.05). Both groups showed a decrease in postoperative VAS and ODI scores (*P* < 0.05); However, there were no significant difference in VAS and ODI scores between the two groups before surgery and at one, three, and 12 months after surgery (*P* > 0.05).

**Conclusions:**

Compared with UBE, OSE has comparable clinical efficacy in treating LDH. Clinical doctors can make reasonable choices based on the specific situation of patients.

## Introduction

Lumbar Disc Herniation (LDH) is a common disease in spinal surgery (1). LDH features are various diseases caused by lumbar degeneration, such as LDH, lumbar spinal stenosis, and lumbar spondylolisthesis (1, 2). The symptoms reported by most LDH patients mainly include lower back pain, lower limb radiating pain, numbness, etc (1–3). These symptoms seriously affect the quality of life of patients.

With the development and application of minimally invasive techniques in clinical practice, percutaneous endoscopic lumbar discectomy (PELD) has gradually become the main treatment for LDH due to its advantages of minimal trauma and fast recovery (4, 5). However, PELD uses a single channel, which poses problems such as narrow surgical field and high recurrence rate (5, 6). Tang et al. (5) reported that PELD treatment for LDH resulted in a reoperation rate of 6.2% (27/435) in patients followed up for two years. Li et al. (6) found that after PELD treatment, the recurrence rate of LDH was as high as 10.38%. In recent years, unilateral biportal endoscopy (UBE) has developed rapidly, with advantages such as wide field of view, flexible operation, sufficient decompression, and high efficiency (7, 8). Jiang et al. (8) confirmed that UBE technology for LDH can produce clinical effects similar to PELD. One hole split endoscope (OSE) is similar to UBE, but it combines dual channels into a single channel, and the instrument and endoscope are separated after passing through the single channel (9). Compared with UBE, OSE reduces the number of surgical incisions, and endoscopes and instruments are not limited by fixed channels, which has certain advantages in terms of synergy and mobility (9, 10). Zhang et al. (10) confirmed that OSE technology has advantages such as minimal trauma and fast recovery. However, there are limited reports on the effectiveness of OSE in treating LDH. This study retrospectively compared LDH patients who received OSE and UBE treatment in our department. Intended to provide reference and guidance for the application of OSE in clinical practice of LDH.

## Materials and methods

This was a single-center retrospective clinical study involving human participants. The study was conducted in accordance with the Declaration of Helsinki and was approved by the Ethics Review Board of Xuzhou Cancer Hospital. No animal experiments were involved in this study.

We retrospectively analyzed 101 patients with single-level LDH who underwent UBE or OSE surgery in our hospital from January 2022 to January 2024. All patients were diagnosed by experienced spine surgeons based on clinical symptoms and imaging findings. Because this was a non-randomized study, patients were allocated to the OSE or UBE group according to the surgical procedure actually performed. The choice between OSE and UBE was made preoperatively through shared decision-making between the surgeon and the patient. The surgeon's recommendation was based mainly on imaging and clinical characteristics, including the operated level, protrusion location, anatomical accessibility, severity of nerve root compression, and the absence of exclusion criteria such as segmental instability, recurrent disc herniation, severe central or lateral recess stenosis, or cauda equina syndrome. Because all included patients had single-level LDH and both procedures were considered technically feasible, patient preference after explanation of the two surgical techniques was also considered.

Inclusion criteria:

- Clinical symptoms of back pain or radiation pain;
- Magnetic resonance or CT images of single level LDH related to symptoms;
- Conservative treatment for more than 12 weeks and failure;
- Follow up for at least 12 months.

Exclusion criteria:

- Segment instability;
- Recurrent LDH;
- Severe central or lateral recess stenosis;
- Cauda equina syndrome.
- Spinal tumors;
- Ankylosing spondylitis;
- Lumbar spine fracture.

All patients underwent lumbar spine dynamic x-ray, CT, and MRI examinations before surgery. All patients were treated by the same surgeon. Diabetes mellitus was not considered an absolute exclusion criterion. All patients underwent routine preoperative evaluation, including assessment of general condition and perioperative surgical risk. Patients with diabetes mellitus were eligible for elective surgery only when their blood glucose was considered adequately controlled according to institutional perioperative management practice. Patients with uncontrolled diabetes, active infection, or poor general condition unsuitable for elective endoscopic surgery were not included.

### OSE

All OSE procedures in this study were performed using the interlaminar approach, and no transforaminal OSE procedures were included. After anesthesia induction, the patient was placed in a prone position on the fluoroscopy operating table with the abdomen suspended. After routine disinfection and draping, the endoscope, radiofrequency electrode, grinding drill, and irrigation system were connected. C-arm fluoroscopy was used to confirm the target segment. Taking left incision surgery as an example, make a longitudinal incision 1.2–1.5 cm long; The entrance is located 1.5 cm outside the spinous process responsible for the horizontal line of the intervertebral space. Cut open the skin, subcutaneous tissue, and deep fascia in sequence, and gradually expand the soft tissue to the bone surface of the vertebral plate for blunt separation. Insert OSE and surgical instruments into the incision and open the perfusion system. Select a total of 3000 mL isotonic saline solution as the flushing solution and place it at a level of approximately 50 cm above the surgical area. After the operative field was irrigated with isotonic saline, the soft tissue in the interlaminar space and the superficial tissue over the ligamentum flavum were cleared using a low-temperature plasma radiofrequency probe until the lower edge of the superior lamina, the upper edge of the inferior lamina, the ligamentum flavum, the root of the spinous process, and the medial edges of the facet joints were exposed. Use high-speed dynamic grinding drill and layered bite device to remove part of the vertebral plate bone to the attachment point of the ligamentum flavum, and separate the adhesion between the dural sac and the ligamentum flavum. Remove the left side of the ligamentum flavum to expose the dura mater, nerve roots, and intervertebral disc. Under endoscopy, nerve retractors are used to protect the dural sac and nerve roots from damage, and a neurodissector is used to peel off adhesions and explore free intervertebral discs and annulus fibrosus. The nucleus pulposus forceps are used to remove protruding and loose nucleus pulposus tissue in the intervertebral space. Correctly remove calcified adhesions around nerve roots to ensure complete decompression. The left nerve root channel and spinal canal further expand. Finally, apply radiofrequency contraction fiber ring. Use bipolar radiofrequency blade to stop bleeding, rinse the field of view with water, implant a silicone drainage tube connected to a drainage bag, pull out the working sleeve, suture the incision, and cover the area with sterile dressing.

### UBE

C-arm fluoroscopy is used to confirm the lower edge of the superior vertebral plate responsible for the intervertebral space. Two transverse incisions approximately 1.2 cm in length are made on the upper and lower sides, with the intersection point of the horizontal line of the lower edge of the vertebral plate and the inner edge of the upper and lower pedicle as the center. The distance between the two incisions is about 3.0 cm. Arrange work and observation channels according to the patient's body type and specific surgical method. The remaining parts of the surgery were performed in the same manner as the OSE group.

Baseline characteristics included age, sex, body mass index (BMI), responsible intervertebral space, and protrusion location. The main clinical outcomes were 12-month VAS-back, VAS-leg, and ODI scores, which were used to evaluate pain relief and functional recovery. VAS-back, VAS-leg, and ODI scores were also recorded preoperatively and at 1, 3, and 12 months postoperatively to assess longitudinal changes. Perioperative variables, including hemoglobin (Hb), hematocrit (Hct), incision length, total blood loss (TBL), surgical duration, hospital stay, complications, and modified MacNab outcomes, were regarded as secondary or supportive outcomes. The modified MacNab criteria included four levels: excellent, good, fair, and poor; excellent and good outcomes were considered clinically satisfactory.

Postoperative recurrence was defined as the reappearance of radicular pain or neurological symptoms after initial postoperative symptom relief, involving the same operated lumbar level, with recurrent disc herniation confirmed by MRI or CT when clinically indicated. All patients were followed for at least 12 months through outpatient visits or telephone follow-up. Follow-up imaging was not routinely scheduled for all asymptomatic patients; instead, MRI or CT was performed when recurrent symptoms or neurological findings suggested possible recurrence. Reoperation for recurrent disc herniation was recorded when performed, but recurrence was not defined solely by reoperation.

This was an exploratory retrospective study. No prior sample size or power calculation was performed. The sample size was determined by the number of consecutive eligible patients who underwent OSE or UBE during the study period and completed at least 12 months of follow-up. Statistical analyses were performed using SPSS 27.0 (IBM SPSS Statistics for Windows, Armonk, NY, USA). The distribution of continuous variables was assessed before analysis. Normally distributed continuous variables were presented as mean ± standard deviation (SD) and compared between groups using independent-samples t tests. Non-normally distributed continuous variables were presented as median and interquartile range (IQR) and compared between groups using the Mann–Whitney U test. Categorical variables were expressed as *n* (%) and compared using the chi-square test or Fisher's exact test, as appropriate. VAS-back, VAS-leg, and ODI scores measured preoperatively and at 1, 3, and 12 months postoperatively were analyzed as repeated-measure outcomes. Repeated-measures analysis was used to evaluate the effects of time, group, and the group-by-time interaction. When longitudinal changes within each group were further examined, paired comparisons between time points were performed with consideration of multiple comparisons. Between-group comparisons at each time point were performed to describe differences between the OSE and UBE groups during follow-up. To reduce the influence of measured baseline confounders related to the non-randomized treatment allocation, additional multivariable linear regression analyses were performed as supplementary sensitivity analyses. For 12-month VAS-back, VAS-leg, and ODI outcomes, the corresponding preoperative score, age, sex, and BMI were included in the models. For postoperative Hb and Hct, the corresponding preoperative value, age, sex, and BMI were included. For TBL and surgical duration, age, sex, and BMI were included as covariates. Results were reported as *β* coefficients with 95% confidence intervals (CIs). Because of the small number of complication events, binary outcomes were compared using Fisher's exact test rather than multivariable logistic regression to avoid model instability. A two-sided *P* value <0.05 was considered statistically significant.

## Results

This study included a total of 101 patients (35 males and 66 females; aged 35–84 years, with a median age of 52 (46–59) years). The OSE group included 48 patients (19 males and 29 females) with a median age of 55 (47.5–59.5) years. The UBE group included 53 patients (16 males and 37 females) with a median age of 51 (45–59) years. The detailed patient characteristics are shown in Table 1. There were no significant difference of age, gender, BMI, responsible intervertebral space, and protrusion position between two groups (*P* > 0.05).

**Table 1:** Comparison of basic characteristics between two groups.

Characteristics	OSE (*n* = 48)	UBE (*n* = 53)	Z/*χ^2^/t*	*P*
Age (years), median (IQR)	55 (47.5–59.5)	51 (45–59)	–	0.344^a^
Female (Yes), *n* (%)	29 (60.4)	37 (69.8)	0.982	0.322
BMI (kg/m^2^), mean ± SD	22.5 ± 3.0	23.3 ± 3.4	−1.305	0.195
Responsible intervertebral space, *n* (%)			3.065	0.216
L3-4	3 (6.3)	7 (13.2)		
L4-5	32 (66.7)	38 (71.7)		
L5-S1	13 (27.1)	8 (15.1)		
Protrusion location, *n* (%)			0.969	0.325
Central type	12 (25.0)	18 (34.0)		
Paracentral type	36 (75.0)	35(66.0)		

Data are presented as mean ± SD, median (IQR), or *n* (%), as appropriate.

UBE, unilateral biportal endoscopy; OSE, one-hole split endoscopy; BMI, body mass index; SD, standard deviation; IQR, interquartile range.

a indicates the Mann–Whitney U test.

As shown in Table 2, Hb and Hct levels decreased after surgery in both groups compared with preoperative values. However, the absolute postoperative Hb and Hct levels were significantly higher in the OSE group than in the UBE group (*P* < 0.05). The incision length and TBL in the OSE group were lower than those in the UBE group, but the surgical time was longer than that in the UBE group (*P* < 0.05). There was no statistically significant difference in hospital stay between the two groups (*P* > 0.05). All patients did not switch to open procedures midway. In the OSE group, one patient with dural tear did not receive special treatment during the surgery because the tear was small. The patient rested in bed for one week after surgery, and the condition remained stable with no cerebrospinal fluid leakage. Two cases of transient sensory decline occurred in the UBE group after surgery, and the symptoms disappeared after receiving nutritional nerve therapy. Both groups had good postoperative wound healing and no infection occurred. To further address potential baseline confounding due to non-randomized treatment allocation, supplementary adjusted analyses were performed (Supplementary Table 1). After adjustment for the corresponding baseline values and measured covariates including age, sex, and BMI, no significant between-group differences were observed in 12-month VAS-back scores (*β* = −0.05, 95% CI: −0.27–0.18, *P* = 0.669), VAS-leg scores (*β* = 0.02, 95% CI: −0.21–0.24, *P* = 0.890), or ODI scores (*β* = −1.59, 95% CI: −4.65–1.48, *P* = 0.307). The OSE group remained associated with lower TBL (*β* = −7.92 mL, 95% CI: −12.58 to −3.26, *P* = 0.001), higher postoperative Hb (*β* = 9.29 g/L, 95% CI: 5.48–13.10, *P* < 0.001) and Hct levels (*β* = 2.68%, 95% CI: 2.31–3.04, *P* < 0.001), and longer surgical duration (*β* = 7.38 min, 95% CI: 2.60–12.17, *P* = 0.003). These adjusted findings were generally consistent with the primary unadjusted results. Under the sample size of this study, no statistically significant difference was observed in the incidence of complications between the two groups (OSE: 1/48, 2.1%; UBE: 2/53, 3.8%; *P* = 1.000; Supplementary Table 2). All patients completed at least 12 months of follow-up. During the follow-up period, no symptomatic and imaging-confirmed recurrence or reoperation for recurrence was identified in either group. According to the modified MacNab criteria, the excellent/good rates were 87.5% in the OSE group and 81.1% in the UBE group; under the sample size of this study, no statistically significant between-group difference was observed (*P* = 0.425; Supplementary Table 2).

**Table 2:** Comparison of perioperative data between OSE group and UBE group.

Index	OSE (*n* = 48)	UBE (*n* = 53)	*t/Z*	P
Preoperative Hb (g/L), mean ± SD	131.8 ± 17.7	133.7 ± 20.2	−0.489	0.624
Postoperative Hb (g/L), mean ± SD	120.6 ± 16.8	113.1 ± 19.1	2.071	0.040
Preoperative Hct (%), mean ± SD	38.9 ± 2.5	39.9 ± 3.2	−1.643	0.100
Postoperative Hct (%), mean ± SD	36.8 ± 2.3	35.0 ± 2.7	3.696	<0.001
Incision length (cm), mean ± SD	1.73 ± 0.43	2.28 ± 0.39	−6.775	<0.001
TBL (mL), mean ± SD	48.4 ± 11.2	56.5 ± 12.1	−3.478	<0.001
Surgical duration (minutes), mean ± SD	65 ± 10.9	57.7 ± 12.5	3.105	0.002
Hospital stay (days), median (IQR)	6 (5–8)	6 (5–7)	–	0.189^a^
Complication, *n* (%)	1 (2.1)	2 (3.8)	–	1.000^b^
Excellent/good rate, *n* (%)	42 (87.5)	43 (81.1)	–	0.425^b^

Data are presented as mean ± SD, median (IQR), or *n* (%), as appropriate.

UBE, unilateral biportal endoscopy; OSE, one-hole split endoscopy; Hb, hemoglobin; Hct, hematocrit; TBL, total blood loss; SD, standard deviation; IQR, interquartile range.

a indicates the Mann–Whitney U test.

b indicates the Fisher's exact test.

**Figure 1:**
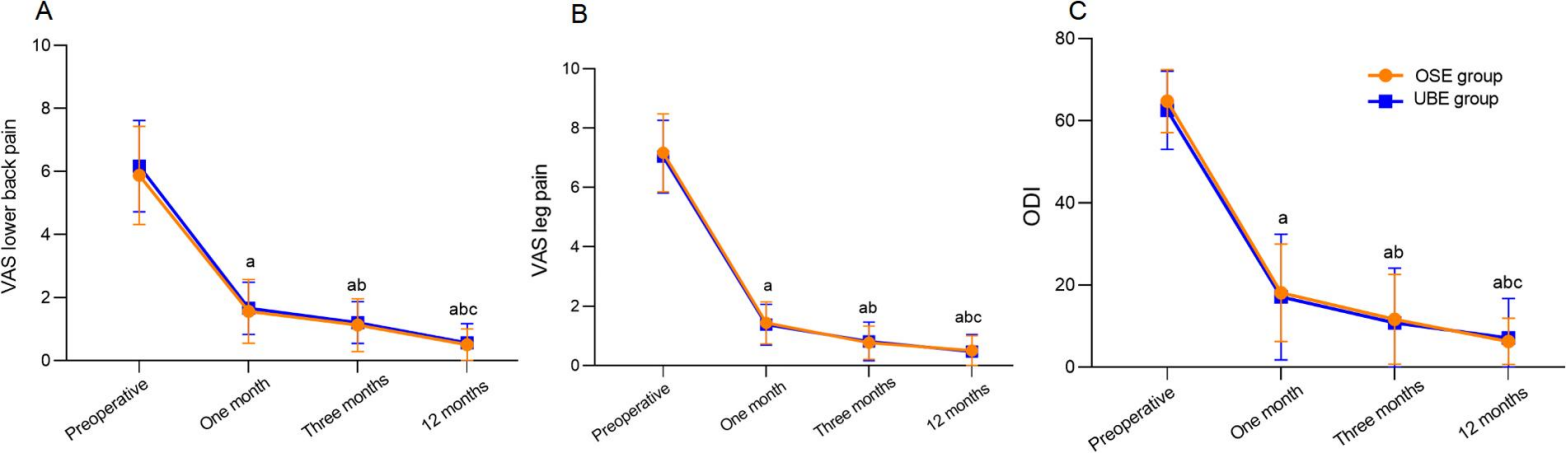
**(A)** Changes in VAS-back scores over time. **(B)** Changes in VAS-leg scores over time. **(C)** Changes in ODI scores over time. Compared with preoperative results in the same group, ^a^*P* < 0.05; Compared with the same group one month after surgery, ^b^*P* < 0.05; Compared with the same group at three months after surgery, ^c^*P* < 0.05. VAS, visual analog scale; ODI, Oswestry disability index.

As shown in Figure 1 and Table 3, both groups showed marked decreases in VAS-back, VAS-leg, and ODI scores after surgery compared with baseline. The improvements continued during postoperative follow-up, and no statistically significant between-group differences were observed at 1, 3, or 12 months after surgery. The between-group mean differences in 12-month VAS-back, VAS-leg, and ODI scores were small, with confidence intervals close to zero and negligible effect sizes. For perioperative continuous variables, Table 3 further showed that the OSE group had a shorter incision length, lower TBL, higher postoperative Hb and Hct levels, and longer surgical duration than the UBE group, while hospital stay did not differ significantly between groups.

**Table 3:** Comparison of clinical outcomes and perioperative continuous variables between the OSE and UBE groups.

Variable	OSE (*n* = 48)	UBE (*n* = 53)	Mean Difference (95% CI)	P	Hedges’ g
Clinical Outcomes					
Preoperative VAS-back	5.9 ± 1.6	6.2 ± 1.5	−0.29 (−0.88, 0.28)	0.478	−0.20
VAS-back at 1 month	1.6 ± 1.0	1.7 ± 0.8	−0.10 (−0.44, 0.27)	0.218	−0.11
VAS-back at 3 months	1.1 ± 0.8	1.2 ± 0.7	−0.08 (−0.37, 0.22)	0.324	−0.11
VAS-back at 12 months	0.5 ± 0.5	0.6 ± 0.6	−0.07 (−0.28, 0.15)	0.713	−0.12
Preoperative VAS-leg	7.2 ± 1.3	7.0 ± 1.2	0.13 (−0.39, 0.63)	0.512	0.10
VAS-leg at 1 month	1.4 ± 0.7	1.4 ± 0.7	0.06 (−0.21, 0.33)	0.522	0.09
VAS-leg at 3 months	0.8 ± 0.6	0.8 ± 0.7	−0.04 (−0.27, 0.19)	0.830	−0.07
VAS-leg at 12 months	0.5 ± 0.5	0.5 ± 0.6	0.03 (−0.18, 0.23)	0.642	0.05
Preoperative ODI (%)	64.8 ± 7.7	62.5 ± 9.5	2.22 (−1.11, 5.50)	0.097	0.25
ODI at 1 month (%)	18.1 ± 11.9	17.1 ± 15.3	1.03 (−4.20, 6.30)	0.154	0.07
ODI at 3 months (%)	11.6 ± 10.9	10.7 ± 13.4	0.89 (−3.78, 5.59)	0.207	0.07
ODI at 12 months (%)	6.2 ± 5.6	7.1 ± 9.6	−0.83 (−3.88, 2.09)	0.377	−0.10
Perioperative Continuous Variables					
Incision length (cm)	1.7 ± 0.4	2.3 ± 0.4	−0.55 (−0.71, −0.39)	<0.001	−1.34
TBL (mL)	48.4 ± 11.2	56.5 ± 12.1	−8.12 (−12.60, −3.57)	<0.001	−0.69
Surgical duration (minutes)	65.0 ± 10.9	57.7 ± 12.5	7.28 (2.65, 11.63)	0.002	0.61
Hospital stay (days)	6.0 (5.0–8.0)	6.0 (5.0–7.0)	0.47 (−0.31, 1.26)	0.189	0.23
Preoperative Hb (g/L)	131.8 ± 17.7	133.7 ± 20.2	−1.86 (−9.31, 5.42)	0.624	−0.10
Postoperative Hb (g/L)	120.6 ± 16.8	113.1 ± 19.1	7.45 (0.57, 14.26)	0.040	0.41
Preoperative Hct (%)	38.9 ± 2.5	39.9 ± 3.2	−0.95 (−2.09, 0.18)	0.100	−0.32
Postoperative Hct (%)	36.8 ± 2.3	35.0 ± 2.7	1.85 (0.89, 2.83)	<0.001	0.73

Data are presented as mean ± SD unless otherwise indicated. Hospital stay (days) is presented as median (IQR). Mean difference was calculated as OSE minus UBE. Effect size is expressed as Hedges’ g.

UBE, unilateral biportal endoscopy; OSE, one-hole split endoscopy; CI, confidence interval; VAS, visual analog scale; ODI, Oswestry disability index; Hb, hemoglobin; Hct, hematocrit; TBL, total blood loss; SD, standard deviation; IQR, interquartile range. For non-normally distributed variables, *P* values were calculated using the Mann–Whitney U test.

## Discussion

The results of this study showed that during the follow-up period, the significant improvements in pain scores and functional status observed in both groups were consistent with previous research findings (10–13). The improved MacNab criteria showed that both the OSE and UBE groups had acceptable rates of excellence at 12 months postoperatively. Indicating that the clinical efficacy of OSE technology is comparable to that of UBE technology. However, the incision length in the OSE group was significantly shorter than that in the UBE group. This fully demonstrates the advantages of single hole minimally invasive OSE technology, where smaller incisions are beneficial in reducing surgical damage to surrounding tissues, lowering postoperative pain, and promoting wound healing. This is consistent with the research results of (13). Meanwhile, the TBL in the OSE group was lower than that in the UBE group. This may be related to the smaller damage to surrounding blood vessels caused by single hole operation of OSE technology. This is consistent with the report by Qing et al. (14). However, the surgery time in the OSE group was longer than that in the UBE group. We believe that the reason may be that OSE technology operates through a single incision, resulting in a relatively narrow surgical space and limited flexibility in instrument manipulation. Doctors need to spend more time adjusting the angle and position of instruments when performing complex operations (9, 13). Related reports also point out that OSE technology poses certain challenges in terms of operating space and instrument flexibility, which may lead to prolonged surgical time (15). UBE technology adopts a dual channel design, with a larger operating space and more flexible instrument operation. In this way, doctors can complete surgical procedures more efficiently, thereby shortening the surgical time (16).

Hb and Hct levels decreased after surgery in both groups, which is consistent with expected perioperative blood loss after endoscopic lumbar surgery. In the present study, the absolute postoperative Hb and Hct levels were higher in the OSE group than in the UBE group. Together with the lower TBL observed in the OSE group, these findings suggest that OSE may be associated with less perioperative blood loss and milder postoperative anemia. This may be related to the smaller incision length and reduced soft-tissue disruption during the single-incision procedure. Similar findings have been reported in previous studies of OSE-related techniques (12, 13, 17, 18).

In this study, there was no significant difference in the incidence of postoperative complications between the two groups. The main reason is that UBE technology has a large operating space and clear field of view, which can reduce the risk of complications during the operation (16). Meanwhile, the experience level of the chief surgeon and the individual anatomical structure of the patient also affect the risk of complications (19). Although the operating space of OSE technology is relatively narrow, the risk of damage to surrounding tissues increases when instruments are operated in a small space. However, skilled surgeons can effectively avoid the occurrence of complications through precise operation (9, 13, 15). In this study, the incidence of complications in both groups was within an acceptable range. Both groups showed substantial postoperative reductions in VAS-back, VAS-leg, and ODI scores, indicating that both procedures were associated with meaningful pain relief and functional recovery. Importantly, the magnitude of postoperative improvement in VAS and ODI exceeded commonly accepted MCID thresholds, suggesting that the improvement was not only statistically significant but also clinically relevant. However, the between-group differences in VAS and ODI scores during follow-up were small, and the corresponding confidence intervals and effect sizes did not suggest a clinically important advantage of either procedure in terms of pain relief or functional recovery. Therefore, under the sample size of this study, OSE and UBE showed similar clinical outcome trends in VAS and ODI improvement. This finding is consistent with previous studies comparing OSE and UBE techniques (13, 14). Although UBE provides a wider working space and greater instrument flexibility through a biportal design, OSE may still achieve adequate decompression through careful endoscopic exposure and precise manipulation. This may explain why the two techniques showed no obvious difference in postoperative pain and functional recovery in this cohort (13, 14, 17, 18).

After 12-month follow-up, there was no significant difference in the excellent and good rates between the two groups based on the modified MacNab criteria evaluation. This once again proves that both UBE and OSE techniques have good therapeutic effects in treating LDH. This suggests that clinical doctors should consider the individual situation of patients comprehensively when choosing surgical methods. For example, the characteristics of the lesion, physical condition, proficiency of the surgeon, economic factors, as well as the advantages and disadvantages of the two techniques. Thus, personalized treatment plans can be developed for patients (20, 21).

This study has several limitations. First, this was a single-center retrospective study with non-randomized treatment allocation. The choice of OSE or UBE was based on surgeon judgment and patient preference, which may have introduced selection bias. Although supplementary adjusted regression analyses were performed to reduce the influence of measured confounders, residual confounding and unmeasured selection bias cannot be completely excluded. Propensity score matching was not used as the main analysis because the relatively small sample size could have led to substantial sample loss and further reduced statistical power. Second, the sample size was relatively small and was not based on a prior power calculation. Therefore, the study may have been underpowered to detect differences in low-incidence outcomes, such as complications and recurrence, and non-significant findings for these outcomes should be interpreted cautiously. In addition, diabetes status and detailed perioperative glycemic parameters were not analyzed separately, which may have limited the assessment of their potential influence on wound healing, infection, and neurological recovery. Third, all procedures were performed by the same spine surgeon. This reduced variability in surgical technique but may limit the generalizability of the findings to other surgeons or centers. Finally, follow-up imaging was mainly symptom-driven rather than routinely scheduled for all asymptomatic patients, which may have limited the detection of asymptomatic radiological recurrence. Other radiological outcomes, such as postoperative lumbar stability and adjacent segment degeneration, were also not systematically assessed. Future multicenter prospective studies with larger sample sizes, predefined power calculations, and more rigorous treatment allocation or matching methods are needed to further validate these findings.

## Conclusion

In this single-center retrospective study, OSE showed similar trends to UBE in postoperative pain relief, functional recovery, and modified MacNab outcomes under the sample size of this study. OSE was associated with shorter incision length, lower TBL, and higher postoperative Hb and Hct levels, but required a longer surgical duration. No statistically significant differences were observed in complications, 12-month VAS/ODI scores, or excellent/good MacNab outcomes between the two groups. These findings suggest that OSE may be a feasible minimally invasive option for selected patients with LDH. However, given the retrospective design and limited sample size, further prospective multicenter studies are needed to confirm these findings.

## Data Availability

The original contributions presented in the study are included in the article/Supplementary Material, further inquiries can be directed to the corresponding authors.
