# Supplementary material for: Comparison of the efficacy of one-hole split endoscopy and unilateral biportal endoscopy in the treatment of lumbar disc herniation
Source: Front Surg. 2026 Jul 21;13:1765537. doi: 10.3389/fsurg.2026.1765537 (PMC13433734; doi:10.3389/fsurg.2026.1765537)
Supplement: Supplementary file 1 [file Supplementaryfile1.docx]

**Supplementary Table 1. Adjusted multivariable linear regression analysis of continuous outcomes**

| **Outcome Variable** | **Unit** | **β coefficient** | **95% CI** | **P** |
| --- | --- | --- | --- | --- |
| VAS-back 12 months | points | -0.05 | (-0.27, 0.18) | 0.669 |
| VAS-leg 12 months | points | 0.02 | (-0.21, 0.24) | 0.890 |
| ODI 12 months | % | -1.59 | (-4.65, 1.48) | 0.307 |
| Postoperative Hb | g/L | 9.29 | (5.48, 13.10) | <0.001 |
| Postoperative Hct | % | 2.68 | (2.31, 3.04) | <0.001 |
| Total blood loss | ml | -7.92 | (-12.58, -3.26) | 0.001 |
| Surgical duration | min | 7.38 | (2.60, 12.17) | 0.003 |

Note: Data are presented as β coefficient (95% CI). β represents the adjusted difference between the OSE and UBE groups, with positive values indicating higher values in the OSE group. UBE was used as the reference group. Models for 12-month VAS and ODI outcomes were adjusted for the corresponding baseline value, age, sex, and BMI. Models for postoperative Hb and Hct were adjusted for the corresponding preoperative value, age, sex, and BMI. Models for TBL and surgical duration were adjusted for age, sex, and BMI.

**Supplementary Table 2. Between-group comparison of binary outcomes**

| **Outcome Variable** | **OSE** | **UBE** | **OR (95% CI)** | **P** |
| --- | --- | --- | --- | --- |
| Complications | 1/48 (2.1%) | 2/53 (3.8%) | 0.54 (0.05, 6.18) | 1.000 |
| Excellent/Good outcome (MacNab) | 42/48 (87.5%) | 43/53 (81.1%) | 1.63 (0.54, 4.88) | 0.425 |

Note: Fisher’s exact test was used for binary outcomes because of the small number of events. OR, odds ratio; CI, confidence interval.
